# Congenital hydrocephalus: new Mendelian mutations and evidence for oligogenic inheritance

**DOI:** 10.1186/s40246-023-00464-w

**Published:** 2023-03-02

**Authors:** Valerie Jacquemin, Nassim Versbraegen, Sarah Duerinckx, Annick Massart, Julie Soblet, Camille Perazzolo, Nicolas Deconinck, Elise Brischoux-Boucher, Anne De Leener, Nicole Revencu, Sandra Janssens, Stèphanie Moorgat, Bettina Blaumeiser, Kristiina Avela, Renaud Touraine, Imad Abou Jaoude, Kathelijn Keymolen, Pascale Saugier-Veber, Tom Lenaerts, Marc Abramowicz, Isabelle Pirson

**Affiliations:** 1 Institut de Recherche Interdisciplinaire en Biologie Humaine et Moléculaire, Université Libre de Bruxelles, Brussels, Belgium; 2 Interuniversity Institute of Bioinformatics in Brussels, Université Libre de Bruxelles-Vrije Universiteit Brussel, Brussels, Belgium; 3 Machine Learning Group, Université Libre de Bruxelles, Brussels, Belgium; 4 Service de Neuropédiatrie, Hôpital Universitaire de Bruxelles and CUB Hôpital Erasme and Université Libre de Bruxelles, Brussels, Belgium; 5 Department of Nephrology, University Hospital of Antwerp, Edegem, Belgium; 6 Human Genetics Department, CUB Hôpital Erasme, Brussels, Belgium; 7 Hopital Universitaire des Enfants Reine Fabiola and Hopital Universitaire de Bruxelles and Université Libre de Bruxelles, Brussels, Belgium; 8 Centre de génétique humaine - CHU de Besançon, Université de Bourgogne-Franche-Comté, Besançon, France; 9 Centre de Génétique Humaine, Cliniques Universitaires Saint-Luc et Université Catholique de Louvain, Brussels, Belgium; 10 Center for Medical Genetics, Ghent University Hospital, Ghent, Belgium; 11 Centre de Génétique Humaine, Institut de Pathologie et de Génétique, Gosselies, Belgium; 12 Center of Medical Genetics, Antwerp University and Antwerp University Hospital, Edegem, Belgium; 13 Department of Clinical Genetics, Helsinki University Hospital, Helsinki, Finland; 14 Génétique Clinique Chromosomique et Moléculaire, CHU de Saint-Etienne, St-Priest-en-Jarez, France; 15 Department of Gynecology and Obstetrics, Abou Jaoude Hospital, Jal El Dib, Lebanon; 16 Center for Medical Genetics, UZ Brussels, Jette, Belgium; 17 Department of Genetics and Reference Center for Developmental Disorders, Université Rouen Normandie, Inserm U1245 and CHU Rouen, Rouen, France; 18 Artificial Intelligence Lab, Vrije Universiteit Brussel, Brussels, Belgium; 19 Department of Genetic Medicine and Development, University of Geneva, Geneva, Switzerland

**Keywords:** Congenital hydrocephalus, Oligogenic inheritance, Exome sequencing, Mutation burden test, Cilia

## Abstract

**Background:**

Congenital hydrocephalus is characterized by ventriculomegaly, defined as a dilatation of cerebral ventricles, and thought to be due to impaired cerebrospinal fluid (CSF) homeostasis. Primary congenital hydrocephalus is a subset of cases with prenatal onset and absence of another primary cause, e.g., brain hemorrhage. Published series report a Mendelian cause in only a minority of cases. In this study, we analyzed exome data of PCH patients in search of novel causal genes and addressed the possibility of an underlying oligogenic mode of inheritance for PCH.

**Materials and methods:**

We sequenced the exome in 28 unrelated probands with PCH, 12 of whom from families with at least two affected siblings and 9 of whom consanguineous, thereby increasing the contribution of genetic causes. Patient exome data were first analyzed for rare (MAF < 0.005) transmitted or de novo variants. Population stratification of unrelated PCH patients and controls was determined by principle component analysis, and outliers identified using Mahalanobis distance 5% as cutoff. Patient and control exome data for genes biologically related to cilia (SYScilia database) were analyzed by mutation burden test.

**Results:**

In 18% of probands, we identify a causal (pathogenic or likely pathogenic) variant of a known hydrocephalus gene, including genes for postnatal, syndromic hydrocephalus, not previously reported in isolated PCH. In a further 11%, we identify mutations in novel candidate genes. Through mutation burden tests, we demonstrate a significant burden of genetic variants in genes coding for proteins of the primary cilium in PCH patients compared to controls.

**Conclusion:**

Our study confirms the low contribution of Mendelian mutations in PCH and reports PCH as a phenotypic presentation of some known genes known for syndromic, postnatal hydrocephalus. Furthermore, this study identifies novel Mendelian candidate genes, and provides evidence for oligogenic inheritance implicating primary cilia in PCH.

**Supplementary Information:**

The online version contains supplementary material available at 10.1186/s40246-023-00464-w.

## Background

Hydrocephalus refers to the abnormal accumulation of cerebrospinal fluid (CSF) within the cerebral ventricles (ventriculomegaly) and/or subarachnoid spaces. Increased intracerebral pressure may cause tissue injury and irreparable brain damage, and hydrocephalus is hence a potentially devastating condition. Strikingly, in spite of decades of surgery for hydrocephalus, consisting mainly of shunting brain ventricular CSF to the peritoneal cavity, it is often unknown whether hydrocephalus is a cause or consequence of brain damage [1].

Congenital hydrocephalus (CH) affects 2–8 in 10,000 live births, with major morbidity and mortality [2]. CH appears very heterogeneous in its causes, with more than half of the cases secondary to hemorrhage, neoplasm, or infection, while epidemiological studies suggest a genetic etiology for up to 40% of CH cases [3]. In a third of genetic cases, hydrocephalus may occur as the sole or main clinical feature [4, 5], which will be defined throughout the manuscript as primary congenital hydrocephalus (PCH). On the contrary, hydrocephalus can occur as part of a syndrome in association to other anomalies, known as syndromic forms of PCH.

Though many genes have been associated with syndromic forms of hydrocephalus, few genes have been reported to cause PCH. X-linked inheritance of PCH has been associated with mutations in L1 cell adhesion molecule (*L1CAM)* [6, 7], and AP-1 complex sigma-2 subunit (*AP1S2)* [8], which encode proteins involved in neuron guidance and function. Autosomal recessive inheritance was observed with mutations in multiple PDZ domain protein (*MPDZ)* [9], coiled-coil domain-containing protein 88C (*CCDC88C)* [10, 11], EMAP like 1 (*EML1)*, and WD repeat domain 81 (*WDR81)* [12]. The encoded proteins are, respectively, involved in synaptic plasticity, dendrite development, mitotic spindle orientation, and endolysosomal trafficking. More recently, de novo mutations were also identified in a set of genes regulating neural progenitor cell fate*,* which account for a diagnostic yield of 8.5% of the studied CH cohort [13, 14]. Nevertheless, despite significant efforts to identify PCH causal genes, Mendelian inheritance is rare, with the majority of cases remaining unexplained.

Recent reports indicate that disorders previously considered as monogenic are in fact caused by mutant alleles at more than one locus [15–19]. Digenic inheritance has been described in diverse pathologies [20], among which brain disorders such as holoprosencephaly and microcephaly [16, 19]. This suggests that PCH, associated with only few clearly monogenic cases, might consist of an oligogenic disorder in at least a subset of patients.

In hydrocephalus animal models, genetic mutations were identified in genes encoding key proteins of motile or primary cilia function. Mutant mice with deficiencies in motile cilia axonemal proteins, such as hydin, dynein axonemal heavy chain 5 (Dnah5), and coiled-coil domain-containing 151 (Ccdc151) [21–23], exhibit hydrocephalus. Nevertheless, in humans, motile cilia defaults lead to primary cilia dyskinesia (PCD), a disorder characterized by chronic respiratory tract infections, *situs inversus*, and infertility, but rarely associated to hydrocephalus [24]. Knock-out mice for genes involved in ciliogenesis such as Cadherin EGF LAG Seven-Pass G-Type Receptor 2/3 (*Ceslr2/3)* [25], Intraflagellar transport 88 (*Ift88)* [26, 27], and Kinesin family member 6 (*Kif6)* [28], all presenting ependymal cell cilia dysfunction, also exhibit hydrocephalus. Interestingly, the involvement of primary cilia defects in hydrocephalus was reported in elipsa (*ift54)* zebrafish mutants [29] as well as in *Ccdc88c* mice mutants [30]. Taken together, these studies suggest a potential role for primary cilium defects in human PCH.

To better understand the genetic landscape of PCH, we studied a cohort of 28 inbred and outbred families. We first performed whole-exome sequencing (WES) analysis and identified novel mutations in known CH genes, as well as in three novel candidate genes. We then addressed possible oligogenic inheritance of PCH.

## Results

We included 28 genetically undiagnosed probands in our study, 9 of which were from consanguineous families and 19 were outbred. A total of 39 subjects were sequenced, including probands, affected siblings and parents (Additional file 1: Table S1). WES analysis provided a molecular diagnosis in 18% of the PCH cohort, i.e., pathogenic or likely pathogenic variants according to ACMG guidelines [31], as well as the identification of three novel candidate genes.

### WES analysis provides a molecular diagnosis in 18% of the PCH cohort, novel mutations are identified in genes associated with CH

#### CRADD

Two siblings of Finnish origin, presenting with macrocephaly (Table 1, Additional file 1: Table S1-15.1; 15.2), displayed a common homozygous mutation in CASP2 and RIPK1 domain-containing adaptor with death domain (*CRADD)* (c.509 G > A p.(Arg170His)), as reported previously [32, 33]. The p.(Arg170His) variant, located in the last exon of *CRADD* gene, was predicted to have a highly damaging effect on protein function, and yielded a combined annotation-dependent depletion (CADD) score [34, 35] of 24, highest compared to all other variants shared by both fetuses. It was present at a low frequency (5.277 × 10^−4^) in the exome aggregation consortium [36] with 63 alleles reported, all heterozygous, in 17 European (Non-Finnish), 2 Other, and 44 European (Finnish) subjects. The variant was absent from 1000G [37], GoNL [38], ESP6500 [39], and our in-house database. The p.(Arg170His) substitution was previously described in a patient presenting an overlapping phenotype of lissencephaly and megalencephaly [40]. This mutation occurs at a highly conserved residue in the death domain of the protein spanning from amino acid residues 116 to 188, which participates to form a complex that activates Caspase2 and trigger apoptosis. The Arg170Cys mutation abolishes CRADD’s ability to activate caspase-2, resulting in reduced neuronal apoptosis, leading to megalencephaly [40]. Interestingly, another patient harboring a missense mutation targeting the same amino acid lissencephaly and megalencephaly in association to hydrocephalus [40]. CRADD/caspase-2 signaling plays an essential role in human cortical architecture, synaptic plasticity, and cognitive function during brain development [32]. Apoptosis is known to be important in human brain development and its impairment has been associated to brain malformations [41, 42]. Indeed, ventriculomegaly can arise from progressive CSF accumulation due to peri-aqueductal neuronal stem cell hyperproliferation [14]. Taken together, these data suggest that the disruption of this pathway could explain the phenotype of this patient.

**Table 1:** Novel mutations in genes associated with hydrocephalus and confirmation of CRADD mutation

Patient ID	Gene	Zygosity	Inheritance	CADD score	Variant classification (ACMG)	Codes for classifying variants	Transcript variant description	Protein	Protein effect	Ethnicity	Ref
21	*ARID1A*	Het	De novo	40	P	PVS1, PS2 and PM2	NM_006015.4:c.6435delG	p.(Glu2145fs*54)	Frameshift	French	
15.1; 15.2	*CRADD*	Hom	AR	24	LP	PS4, PM2 and PP3	NM_003805.3:c.509G > A	p.(Arg170His)	Missense	Finnish	[32, 40]
16.1; 16.2; 16.3	*KIDINS220*	Hom	AR	NA	LP	PS3, PM2, PM4 and PP1	NM_020738.2:c.2137_2145del	p.(Gln713_Leu715del)	Deletion	Pakistani	[43]
26	*POMGNT1*	Hom	AR	22.8	LP	PVS1 and PM2	NM_001243766.1:c.1539 + 1G > A	p.?	Splicing effect	Belgian	
17	*POMT2*	Hom	AR	24.8	LP	PVS1 and PM2	NM_013382.5:c.333 + 1G > A	p.?	Splicing effect	Moroccan	

The mutations are named according to HGVS nomenclature recommendation, with RefSeq identifier. All mutations are either absent in the homozygous state (CRADD, POMGNT1) or in both heterozygous and homozygous states (ARID1A, KIDINS220, POMT2) from ExAC and gnomAD databases. AR: autosomal recessive; Het: heterozygote; Hom: homozygote; NA: not applicable; P, pathogenic; LP, likely pathogenic; PVS1, null variant (nonsense, frameshift, canonical + / − 1 or 2 splice sites, initiation codon, single or multi-exon deletion); PS2, de novo (both maternity and paternity confirmed) in a patient with the disease and no family history; PS3, well-established in vitro or in vivo functional studies supportive of a damaging effect on the gene or gene product; PS4, prevalence of the variant in affected individuals is significantly increased compared to the prevalence in controls; PM2, absent from controls (or at extremely low frequency if recessive) in Exome Sequencing Project, 1000 Genomes or ExAC; PM4, protein length changes due to inframe deletions/insertions in a non-repeat region or stop-loss variants; PP1, co-segregation with disease in multiple affected family members in a gene definitively known to cause the disease; PP3, multiple lines of computational evidence support a deleterious effect on the gene or gene product (conservation, evolutionary, splicing impact, etc.). Classifications and codes following Ellard et al. 2020[58]

##### KIDINS220

In one family of consanguineous origin, three fetuses (Table 1, Additional file 1: Table S1-16.1; 16.2; 16.3) displayed ventriculomegaly and limb contractures. WES of affected fetuses and both parents revealed a shared homozygote mutation in kinase D interacting substrate 220 (*KIDINS220)* c.2137_2145delCAAGTGCTG; p.(Gln713_Leu715del) which segregated with the phenotype, as reported elsewhere [43]. The three amino acid inframe deletion was absent from 1000G [37], GoNL [38], ESP6500 [39], and our in-house database as well as in gnomAD [44]. The aforementioned glutamine, valine, and leucine residues are highly conserved among mammals, and fall in the binding region of Trka, an NGF receptor which triggers differentiation and survival pathways [45]. Moreover, the presented phenotype was previously reported in one family with three affected siblings, for which pathogenicity was attributed to a homozygous *KIDINS220* mutation [46].

##### ARID1A

First trimester ultrasound revealed hydrocephalus with dilatation of 3rd ventricle, confirmed by foetopathological exam which additionally described aqueductal stenosis and corpus callosum agenesis. By WES trio analysis, we identified a de novo frameshift mutation in AT-rich interaction domain 1A (ARID1A) (c.6435delG; p.(Glu2145fs)) (Table 1, Additional file 1: STable 1–21), absent from 1000G [37], GoNL [38], ESP6500 [39], our in-house database, and gnomAD [44]. Sanger sequencing of the affected fetus confirmed the presence of the frameshift, and its absence in both parents. *ARID1A* encodes a member of the SWItch/Sucrose Non-Fermenting (SWI/SNF) complex, mediating processes such as the regulation of gene expression, cellular proliferation, apoptosis, differentiation, and the repair of genetic material [47]. Recently, a conditional *Arid1a* KO mouse model showed that pancortical *Arid1a* deletion led to extensive mistargeting of intracortical axons and corpus callosum agenesis [47]. Human mutations in this gene are associated with Coffin–Siris syndrome (CSS), a disorder rarely linked to hydrocephalus. Though malformations such as corpus callosum agenesis have been described in CSS caused by pathogenic variants in *ARID1A*, prenatal anomalies are rare with almost all CSS patients ascertained in postnatal period [48]. However, novel fetal findings in association with pathogenic *ARID1A* variants recently reported, overlap the clinical presentation of our fetus (i.e., lung lobulation defects) [48]. Interestingly, the involvement of *ARID1A* in cancer is transposed as a putative mechanism to explain the brain malformations associated to CSS. In cancer, pathogenic *ARID1A* mutations affect subunits of the SWI/SNF complex inducing a disruption in phosphatase and tensin homolog (PTEN) and phosphatidylinositol-4,5-bisphosphate 3-kinase catalytic subunit alpha (PIKC3A) signaling, thus aberrant expression of these genes could lead to brain malformations, as both PTEN and PIKC3A haploinsufficiency have been linked to ventriculomegaly/hydrocephalus [48].

###### POMT2 and POMGNT1

Two unrelated fetuses presented at the time of evaluation (undisclosed information and 22 weeks of gestation, respectively) with severe ventriculomegaly. In each proband, a homozygous mutation was found (Table 1, Additional file 1: Table S1-17, 26) in genes encoding proteins involved in O-mannosylglycan biosynthesis. In the first fetus, WES analysis uncovered a homozygote mutation in protein O-mannosyltransferase 2 (*POMT2)* c.333 + 1 G > A, while in the second fetus a homozygote mutation in protein O-linked mannose N-acetylglucosaminyltransferase 1 (*POMGNT1)* c.1539 + 1 G > A was found. Both mutations were predicted pathogenic with respective pathogenic CADD scores of 24.8 and 22.8, and resided in splice site donors, which in general leads to single exon skipping [49]. In *POMGNT1,* the impacted exon lies in the proteins catalytic region, while in *POMT2* it lies within the transmembrane helices of the protein. *POMT2* and *POMGNT1* are involved in two clinically similar disorders, respectively Walker–Warburg syndrome (WWS) and muscle–eye–brain disease (MEB) [50, 51]. Both are linked to a severe neuronal migration disorder associated with hydrocephalus and muscular dystrophy, though the physiopathology is usually less severe in MEB [52–55]. Several studies indicate that O-mannosylation of alpha-dystroglycan (⍺-DG), a highly glycosylated surface membrane protein, plays an important role in muscle and brain development [50, 56]. Indeed, in WWS patients, the highly glycosylated ⍺-DG was selectively deficient in skeletal muscle and brain [57].

### Three new potential candidate genes revealed by WES analysis

#### RNPC3

**Fig. 1:**
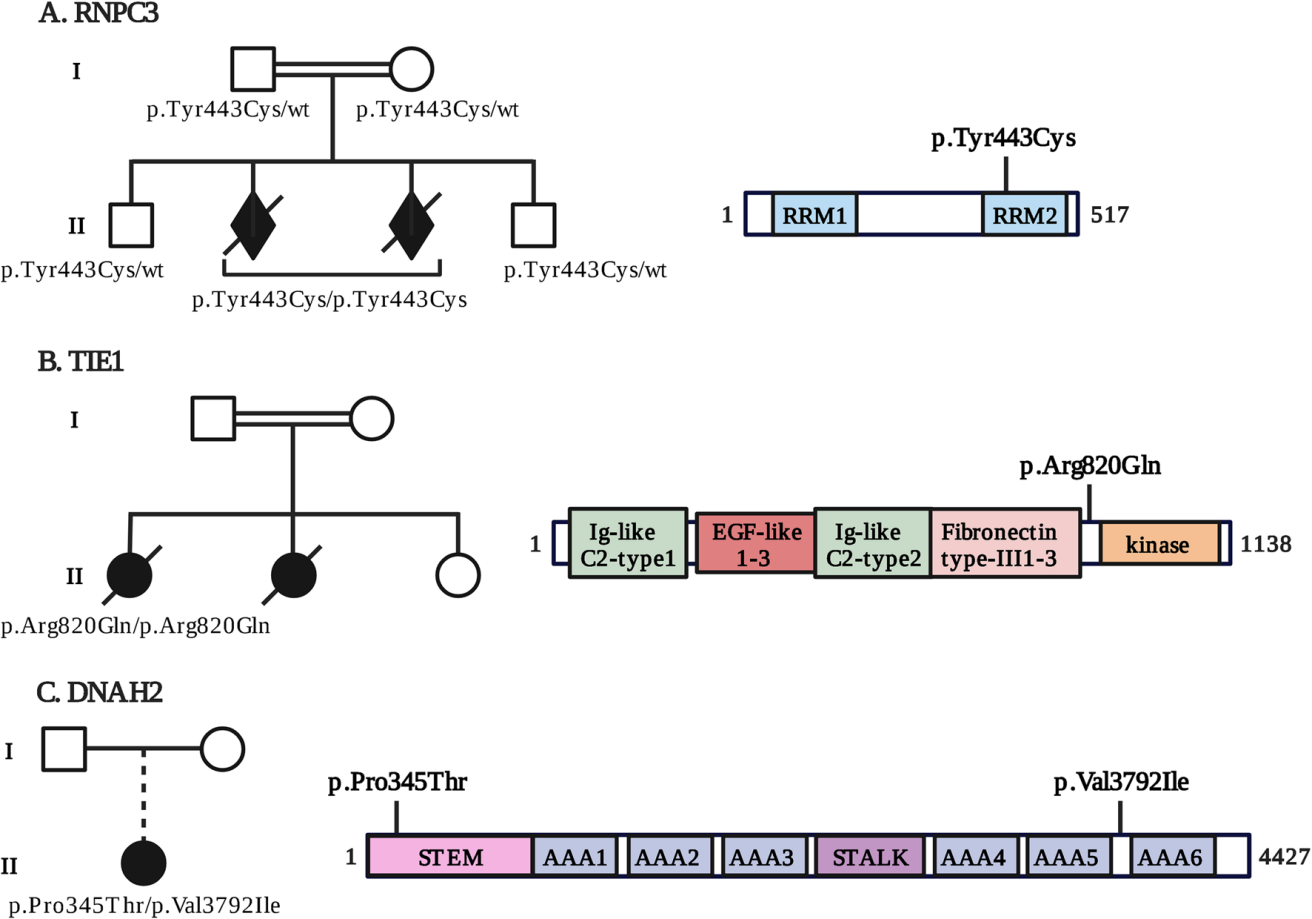
Schematic representation of the three potential candidate genes and their respective family tree. Family trees showing proband 28.1 (**A**), proband 10 (**B**) and proband 22 (**C**), and their respective genotypes. The dotted line represents adoption. Schematic representation of the proteins’ domains and position of variants in candidate genes are indicated. Image created with BioRender.com

In a consanguineous family of Turkish origin, a homozygous missense variant in RNA binding region containing 3 (*RNPC3)* c.1328A > G; p.(Tyr443Cys) was identified in two affected fetuses (Fig. 1A, Additional file 1: Table S1-28.1; 28.2). Familial segregation was confirmed by Sanger sequencing. The variant falls into a homozygote stretch of 12 Mbp ranging from position chr1: 100.984.092–113.000.946. The variant was not reported in gnomAD [44], and the exon harboring the mutation is predicted intolerant to variation. The variant was predicted deleterious with a CADD score of 16.94. Interestingly, a recent article describes a patient with severe growth delay, and anatomic brain anomalies including an enlargement of the peri-cerebral spaces, in which the same *RNPC3* variant was identified [59] The tyrosine residue is a well-conserved amino acid in the RNA recognition motif 2 (RRM2) involved in the binding of RNPC3 to small nuclear RNAs. Indeed, *RNPC3* gene encodes for a component the pre-mRNA splicing machinery, the minor (U12-dependent) spliceosome complexes, reported to target around 700–800 genes [60].

#### TIE1

A homozygote variant in tyrosine kinase with immunoglobulin like and EGF like domains 1 (*TIE1)* c.2459 G > A; p.(Arg820Gln) was identified in an consanguineous family (Fig. 1B, Additional file 1: Table S1-10). The variant was absent from all data databases, and was predicted to yield a damaging effect on the protein. Harboring a CADD score of 32, this variant was the most deleterious homozygote variant identified in the patient. Indeed, bioinformatic predictions (https://www.mutationtaster.org/;https://hsf.genomnis.com/; http://wangcomputing.com/assp/) show that the missense mutation could introduce a new splice site, affecting RNA splicing.

#### DNAH2

In one patient (Fig. 1C, Additional file 1: Table S1-22) presenting with triventricular hydrocephalus, a brain cyst, encephalocele, and an incomplete cerebellum, we identified a compound heterozygote variant in dynein axonemal heavy chain 2 (*DNAH2),* encoding a heavy chain subunit of the inner dynein arm-f (dynein f), a component of motile cilia [61]. The NM_020877.2: c.2493C > A; p.(Pro345Thr) and c.12834G > A; p.(Val3792Ile) variants harbored respective CADD scores of 26 and 15, and both were absent from 1000G [37], GoNL [38], ESP6500 [39], and our in-house database. The first variant p.(Pro345Thr) lies within the STEM domain of the protein involved in interaction with other dynein components. DNAH2 is part of the axonemal inner dynein arm complex and plays a central role in ciliary beating [61]. Notably, compound heterozygote variants (NM_020877.2: c.2190C > T, p.(Arg244Trp) / c.7192G > C, p.(Gly1911Ala); c.3246C > T, p.(Arg596X) / c.4696A > G, p.(Asp1079Gly)) were found in two different probands presenting primary microcephaly [62].

### Oligogenic inheritance: patients with PCH display an excess of variants in primary cilia genes

Although WES analysis rendered a molecular diagnosis for 18% of our cohort, a significant number of cases remained unsolved, for whom we suspected non-Mendelian modes of inheritance. Many animal models which present hydrocephalus as main feature are models in which motile cilia genes are impaired [63, 64], though the impairment of the same group of genes in humans is responsible for PCD, a ciliopathy rarely associated with hydrocephalus [65, 66]. Conversely, there is some recent evidence linking primary cilia defects and hydrocephalus [29].

**Fig. 2:**
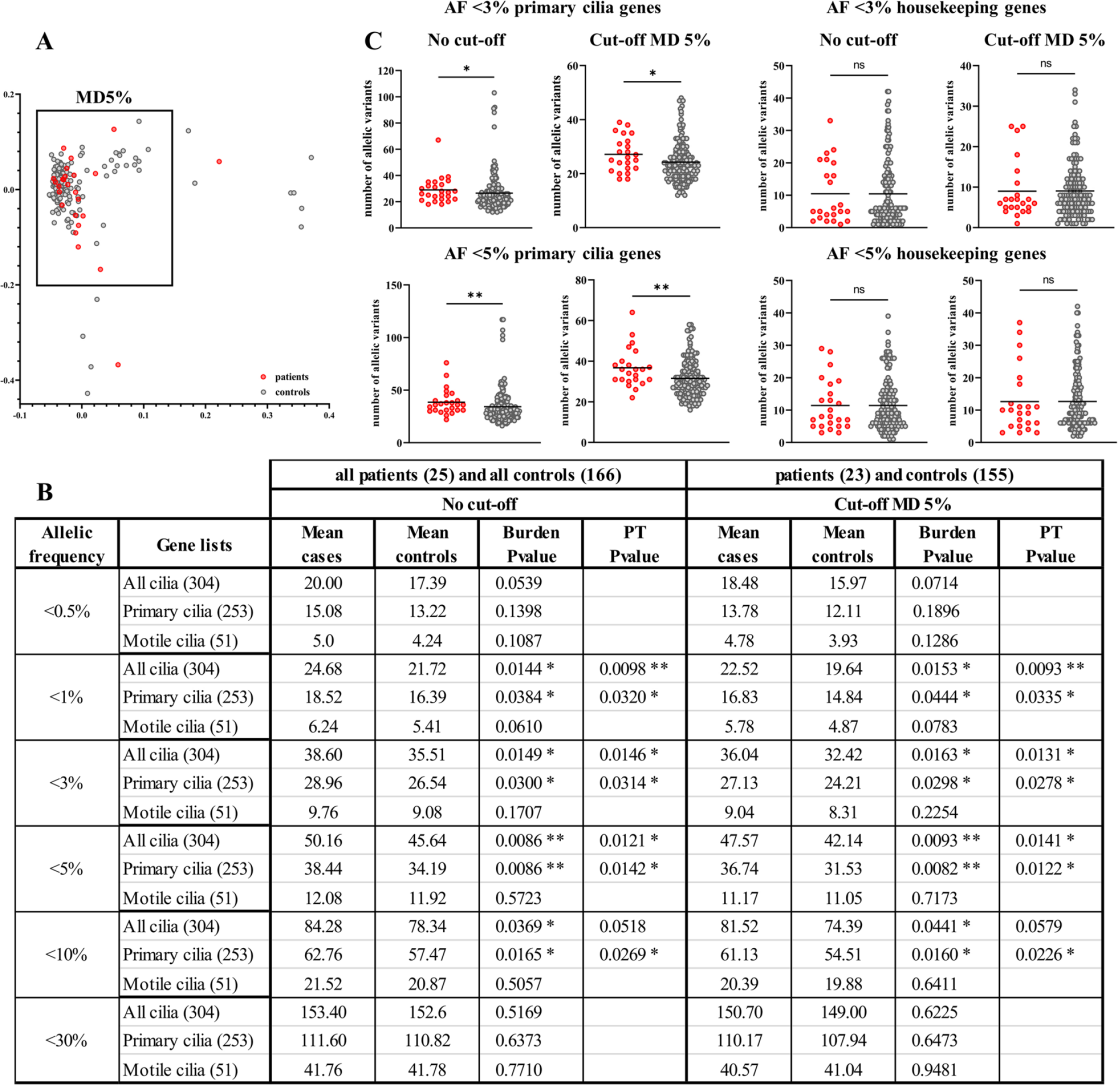
PCH patients display a burden of variants in primary ciliary genes. **A** Plot represents the distribution of patients and controls used in mutation burden test analysis. Each dot represents a sample (exome sequencing data) and each color represents a type of sample: patients (red circles) and controls (gray circles). The square englobes the subset of patients and controls determined by MD5% cutoff. **B** Calculated p values by Wilcoxon test for mutation burden tests (Burden p value) and on 10,000 permutation test (PT p value) at different allelic frequencies (AFs) with or without cutoff at MD5%. *p value ≤ 0.05, **p value ≤ 0.01. **C** (upper panels) Variants in 253 primary cilia genes (left) and in 253 housekeeping genes (right) identified via exome sequencing filtered for AF < 3% in patients and controls with or without MD5% cutoff. (Lower panels) Variants in 253 primary cilia genes (left) and in 253 housekeeping genes (right) identified via exome sequencing filtered for AF < 5% in patients and controls with or without MD5% cutoff

We therefore compared the burden of variants in ciliary genes, obtained from the SYScilia database [67], between our patient and control groups. This analysis was conducted using the complete gene list comprised of 304 genes, and on a subdivision of the latter (in two subsets of genes linked to motile and primary cilia). The variants were filtered as described in the Methods section, for various allelic frequencies (AFs < 0.5, 1, 3, 5, 10, and 30%). Principal component analysis (PCA) was used to identify potential outliers in 25 unrelated PCH patients and 166 control patients with non-neurological disorders based on the Mahalanobis distance (MD). The MD is a distance metric that allows inference of the distance of a point with regard to a multivariate distribution (PCA) while employing the covariance structure [68]. Here, we used a robust version of MD which computes the distance (in terms of deviation from the multivariate centroid) for each data point and we set a cutoff value based on the distribution of those distances [69]. The points with distances greater than the cutoff are deemed to be outliers. MD with a significance level of 5% (MD5%) was applied to the two first principal components of the PCA, meaning that 5% of the most extreme data points were considered as outliers. This analysis conserved 23 patients and 155 controls (Fig. 2A) and excluded 2 patients and 11 controls as they were considered as outliers.

The mutation burden test revealed a statistically significant burden in PCH patients in ciliary genes and more particularly in primary ciliary genes, over a range of AF (< 3%, < 5%), consistent with oligogenic inheritance (Fig. 2B). Interestingly, we maintain significance for AF < 10% in primary cilia. The smallest p value (p = 0.0082) was observed in the primary cilia subset of genes at an AF < 5% with MD5% cutoff (Fig. 2B, C, lower right panel).

The same trend was maintained in the cohort prior to PCA analysis, where PCH patients displayed a higher number of allelic variants in the primary cilia genes for AF < 3% and above (Fig. 2B).

As a control, we measured the burden of allelic variants in housekeeping genes among PCH and control patients for all conditions which gave a significant p value and observed no significant difference between the two groups (Fig. 2C, right panels). To exclude the effect of chance in the selection of the ciliary genes, 10,000 permutations were performed with either 304 or 253 randomly chosen housekeeping genes (Additional file 1: Figure S1). A significance level of *α* = 0.05 was considered, with an expected value of less than 500 subsets of housekeeping genes resulting in a significant Wilcoxon statistics (10,000 trials * 0.05). The number of subsets of housekeeping genes yielding a Wilcoxon statistic with a smaller p value than for the ciliary genes was counted (Additional file 1: Figure S2). Respectively, only 278 (AF < 3%) and 122 (AF < 5%) random selections of housekeeping genes resulted in a significant p value with MD5% (Fig. 2B). In other words, only 2.8% and 1.2% of randomly chosen lists harbor a smaller p value compared to the corresponding burden test p value obtained with the ciliary gene lists. The same analysis conducted on the total cohort gave 314 and 142 random selections of housekeeping genes with a significant p value (Fig. 2B). These results allow us to validate with a probability of 95% that the burden of variants observed in our PCH patients is not due to random gene selection.

### Digenic viewpoint: VarCoPP analysis

Finally, patients’ results were re‐examined individually, from a digenic viewpoint, in search of potential cases of pathogenic combinations of two or more variants. A search for common digenic pairs among hydrocephalus patients was also conducted, but did not uncover any conclusive results, possibly due to the modest size of the cohort. The pathogenic pairs in the 99% confidence interval obtained by VarCoPP for each individual patient were evaluated using bioinformatic databases such as PubMed, OMIM, and ClinVar. In addition, we took advantage of the ORVAL [70] platform to explore the predicted digenic effect of the pathogenic pairs. As a result, a candidate pair composed of primary ciliary genes was predicted to have a digenic effect (Additional file 1: Table S2).

The candidate pair is predicted as true digenic by the digenic effect predictor [71] in ORVAL, meaning that the presence of variants in both genes is required to trigger the disease phenotype. The first variant falls in the Intraflagellar transport 172 (*IFT172)* gene (p.Asp907Asn), and is absent from 1000G [37], GoNL [38], ESP6500 [39], and our in-house database as well as in gnomAD [44] and harbors a CADD score of 25.5. The second variant composing the pathogenic pair in tetratricopeptide repeat domain 21B (*TTC21B*) (p.Pro753Leu) was also absent from all aforementioned databases and harbors a CADD score equal to 23.3. IFT172 is part of the IFT-B complex and TTCB21 of the IFT-A complex, respectively, responsible for anterograde and retrograde intraflagellar transport [72], necessary for the structure and functional integrity of the cilium.

## Discussion

In this study, we report the analysis by WES of a PCH cohort of 28 families, 9 of which were inbred and 19 outbred. Almost half of the PCH probands included in this study were from families where two or more cases had been described (13 families), which increased the likelihood of a genetic contribution.

A molecular diagnosis following Mendelian inheritance was found in four families with novel mutations in known genes: *KIDINS220*, *POMT2*, *POMGNT1* and *ARID1A* (Table 1). In one sibship, we identified a previously described homozygous mutation in *CRADD* [40] (Table 1). In some patients who presented prenatally with ventriculomegaly, we identified variants in genes for which hydrocephalus was already reported but as a postnatal feature of a complex syndrome. Some of these cases revealed a posteriori to consist of syndromic associations with hydrocephalus, though ventriculomegaly appeared at the time as the major echographic finding. Indeed, hydrocephalus, albeit the hallmark for the diagnosis of WWS, appears on the prenatal ultrasound mostly in the third trimester [73]. Cases as these reflect the need to include such patients in routine syndromic hydrocephalus genetic screening. Other cases underscore the importance to better describe prenatal features of well-known postnatal phenotypes, e.g., *ARID1A*. This effort will be important to validate which genes should be included in, or excluded from, diagnostic panels for fetal medicine.

Furthermore, WES analysis allowed the identification of apparently Mendelian mutations in three new candidate genes, *RNPC3, TIE1* and *DNAH2,* each of these belonging to cellular processes previously linked to human or mice PCH phenotype, and ubiquitously expressed in brain, albeit with lower expression levels for *DNAH2 (*https://www.proteinatlas.org*)*.

The tyrosine residue variant in *RNPC3* p.(Tyr443Cys) is a well-conserved amino acid in the RNA recognition motif 2 (RRM2) involved in binding small nuclear RNAs. RNPC3 is a component of one of the minor (U12-dependent) spliceosome complex, reported to act on 700–800 RNAs [60]. A zebrafish mutant, caliban (*clbn*), harboring a splicing mutation in the same RRM2 domain of rnpc3, leads to a severe and pleiotropic phenotype in developing zebrafish larvae with early lethality [74]. The authors showed that several genes involved in various steps of mRNA processing, including transcription, splicing, and nuclear export are disrupted in *clbn* mutants. Interestingly, of the 38 downregulated genes, 4 are associated with human pathologies wherein hydrocephalus is a feature [74]. Though in human [75] and mice models [76, 77] defects in minor spliceosome components are associated with several disorders characterized by microcephaly and dwarfism, a recent report describes a case of severe ventriculomegaly and mild growth retardation associated with compound heterozygote mutations in the non-coding region of RNA, U4atac Small Nuclear (*RNU4ATAC)* [78]. In parallel, a recent study in 9 outbred families identified mutations in spliceosome genes peptidylprolyl isomerase like 1 (*PPIL1)* and pre-RNA processing-17 (*PRP17)* causing neurodegenerative pontocerebellar hypoplasia with microcephaly, where one of the probands presented both microcephaly (-4SD) and hydrocephalus[79]. Taken together, these data support *RNPC3* as a likely candidate for PCH.

Exome analysis of a second consanguineous family revealed a homozygote variant in *TIE1*, predicted to introduce a new donor splice site resulting in aberrant RNA splicing. This angioprotein receptor plays a critical role in angiogenic events such as blood vessel homeostasis and endothelial cell survival and in lymphangiogenesis [80]. The major impact in *Tie*^−/−^ mice is on the formation of lymphatic vasculature, with embryos also presenting hemorrhage, both causing death during gestation [81]. In zebrafish, decreasing the expression of *tie1* mRNA correlates with significantly increased eye size and ventricular space [82]. Moreover, defects in angiogenesis components such as FLVCR heme transporter 2 (*FLVCR2)* and Vascular endothelial growth factor (*VEGF)* in humans have been linked to hydrocephalus [79, 83, 84].

To date, autosomal recessive mutations in *DNAH2* have been associated morphological abnormalities of the sperm flagella responsible of male infertility [85]. Depletion of either *Dnah2* or WD repeat domain 78 (*Wdr78)*, another dynein f subunit, by RNAi in mouse ependymal cells resulted in increased incidence of paralyzed motile cilia, and interestingly, knockdown *wdr78* zebrafish larvae displayed hydrocephalus [61]. Moreover, in 2016 Ha et al. characterized two hydrocephalus mouse mutants by WES after whole-genome SNP mapping and revealed novel recessive mutations in two genes encoding for structural components of the motile cilia axoneme, dynein axonemal assembly factor 1 (Dnaaf1) and leucine-rich repeat-containing protein 48 (Lrrc48) [86].

Through WES analysis, Mendelian mutations were found in a minority of PCH probands in our cohort (18%), leaving the majority of cases unexplained. From this observation and the fact that animal models are compatible with a more complex heredity, we hypothesized that missing heritability of PCH could, at least in a number of cases, lie within oligogenic inheritance. In double transgenic mouse model *Tg(Lmo3;Hen2)*, 15% of either *Tg(Lmo3)* or *Tg(Hen2)* pups developed hydrocephalus, whereas all double heterozygote pups presented with hydrocephalus [87]. Moreover, increasing evidence in animal models shows implication of ciliary genes, responsible for cilia biogenesis/maturation, in hydrocephalus. To study the implication of ciliary genes under an oligogenic model as an underlying cause of PCH, we performed mutation burden tests and provide evidence of such inheritance implicating ciliary genes. After clustering analysis using PCA and a MD5% cutoff, WES data of 23 unrelated PCH patients and 155 controls were compared by burden test of variants in ciliary genes in both motile and primary ciliary genes.

We observed a statistically significant mutation burden in PCH patients found over a range of allele frequencies, particularly in primary cilia genes, suggesting that the primary cilium could be an essential component in hydrocephalus pathogeny. The major roles of primary cilia include sensory perception, signal transduction, and cell cycle progression.

Recent key observations showed that protein products of genes mutated in murine hydrocephalus are localized to the primary cilium. For example, defaults in centrosomal protein Cep290 or intraflagellar transport (IFT) components such as Kinesin family member 3a (Kif3a), Ift188 and Ttc21b, impair primary cilia formation/signaling in turn disrupting ependymal cilia and leading to hydrocephalus in mice [88–90]. Moreover, cilium-less radial glia conditional mutants display increased mTOR signaling which leads to enlarged apical domains of radial glial cells (RGCs) and subsequent dilatation of brain ventricles [91]. More recently, deletion of Gpr161 cilia-localized G-protein coupled receptor in mouse neuroepithelial cells and RGCs at early mid-gestation-induced derepression of Sonic Hedgehog (SHH) signaling, leading to hydrocephalus at birth [92]. Finally, the primary cilium is essential in planar cell polarity (PCP), allowing establishment of a polarity axis which organizes cells in the plane of the tissue [93–95]. PCP is also essential for tissue homeostasis [96] and the directional beating of motile cilia. The *Ccdc88c* mice model, with the loss of PCP of ependymal cells, leads to abnormal ependymal flow and hydrocephalus [30]. Indeed, the correct positioning of the primary cilium through translational polarity, at the apical surface of the cell, is mandatory for the correct establishment and proper beating of the motile cilia [97].

We used ORVAL [70], a novel web platform which predicts the potential pathogenicity of an individual’s oligogenic variant combinations. One patient was identified with a true digenic combination *IFT172-TTC21B,* within the 99% confidence interval. Both genes encode components of the IFT complex, B and A, respectively. The high CADD scores of both variants as well as their close biological distance could explain the high disease-causing confidence generated by ORVAL. These results are reinforced by the absence of the same variant combination in the 155 controls and the association of both genes in mutated animal models with hydrocephalus. Both mice and zebrafish animal models carrying an Ift172 mutation displayed hydrocephalus. Indeed, a recessive N-ethyl,N-nitrosurea (ENU)-induced hypomorphic mutation in Ift172 in mice caused VACTERL syndrome associated with hydrocephalus [98], and an ift172 knockdown zebrafish model displayed anomalies including ventral body curvature and hydrocephalus [99]. In a mice model where Ttc21b was ablated in brain and surrounding domains, embryos displayed an enlarged forebrain and ventriculomegaly of the lateral ventricles [89]. Hence, we suggest that the predicted pathogenic combination could explain the observed phenotype in this sibship.

## Conclusion

In conclusion, we report novel mutations in known hydrocephalus genes in 18% of our PCH probands and propose three novel candidate genes: *DNAH2*, *TIE1* and *RNPC3* in a further 11%*.* Mutations in genes known for postnatal, syndromic hydrocephalus presented as isolated PCH in some of our probands. We furthermore report evidence of oligogenic inheritance implicating the primary cilium as an important player in PCH. In one patient, we identified a true digenic combination where both gene products are implicated in intraflagellar transport in primary cilia and, when mutated in animal models, are associated to hydrocephalus. Our data will contribute to identifying novel Mendelian genes; including or excluding genes from clinical diagnostic panels in fetal medicine; and precising the role of primary cilia in brain developmental disorders.

## Patients and methods

### Patient cohort collection

The study was approved by the Department of Scientific Research and Ethics Committee of Erasme Hospital in Belgium under the reference P2019/056. Written informed consent from the patients or legal representatives was obtained. The probands of these families were referred to us by national and international genetic or obstetric departments, based on the following inclusion criteria: primary congenital hydrocephalus without *L1CAM* mutation, abnormal karyotype, or known syndromes (Supplementary Table 3). Included probands presented with PCH that was either isolated or associated with brain malformations. CGH array and/or karyotype, as well as *L1CAM* sequencing were performed in all patients before referral. Medical history, clinical and radiological assessments were obtained by the referring physicians. We included 28 families in our study, 9 of which were inbred and 19 were outbred, and performed a total of 39 exomes (28 probands and 11 relatives) (Supplementary Table 1).

### Preparation of gDNA and whole-exome sequencing

Genomic DNA was extracted from either cultured amniotic fluid cells or from umbilical cord cells. DNA of unaffected relatives was extracted from peripheral blood. Patients’ genomic DNA was sheared and exonic sequences were captured using a DNA capture kit. For WES, DNA samples were prepared in Illumina libraries and then underwent whole-exome enrichment with the NimbleGen Seqcap EZ v3, Agilent SureSelect All Exon v1 and Agilent SureSelect All Exon v5. DNA sequencing platforms varied according to the time of the analysis. Two sequencing platforms were used: AROS applied biotechnology, Denmark (Illumina HiSeq 2000) and BRIGHTcore Brussels Interuniversity Genomics High Throughput core, Brussels, Belgium (Illumina HiSeq 1500).

### Variant classification

Dry-lab processing, base calling of the raw sequencing data, primary sequence analysis and variant calling was performed at the Interuniversity Institute of Bioinformatics in Brussels ((IB)^2^; Brussels, Belgium). In brief, raw sequences were aligned to the reference genome GRCh37 using BWA algorithm version 0.7.15 [100], duplicated reads were then marked using Picard version 1.97 [101], alignment quality was improved using the GATK [102] realigner and base recalibrator version 2.7 and finally, variants were called using GATK Haplotype Caller version 2.7. The resulting variant set was annotated and filtered using the Highlander software (https://sites.uclouvain.be/highlander/index.html). Variants were filtered for quality criteria (pass GATK standard filter, read depth > 5, variant confidence by depth ≥ 10), allelic frequency (AF) < 0.5% (based on the maximum minor AF found in ExAC [36], 1000G [37], ESP6500 [39], gonl [103], ARIC5606 [104] and our in-house database), nonsynonymous or splice junction effect in protein coding genes (using biotype from Ensembl [105] and snpeff_effect from SnpEff [106]), and genotype (homozygous or heterozygous variants). Variants were then sorted by decreasing combined annotation-dependent depletion (CADD) score and consensus score, corresponding to a combined pathogenicity score from the six different predictors included in the Highlander software. 1 point was given for each of the six prediction software (Mutation Taster, Sift, Polyphen2, LTR, Mutation Assessor, FATHMM) when the variant is predicted to be pathogenic. Additional points were given based on type of mutation with the highest score given to frameshift and nonsense predicted effect (snpeff_effect). Variants were inspected manually based on scientific literature and genome databases data, and variant curation followed the American College of Medical Genetics and Genomics (ACMG) guidelines. Variants of interest were then confirmed by Sanger sequencing and familial segregation was undertaken when possible. Possible candidate genes have been submitted to the online Matchmaker exchange platform.

### Sanger sequencing

ExonPrimer software (http://ihg.helmholtz-muenchen.de/ihg/ExonPrimer.html) was used for PCR primer design (Supplementary Table 4). All exons and flanking intronic regions of the candidate genes were sequenced by the Sanger method using the Big Dye Terminator cycle sequencing kit v2 (Applied Biosystems, Foster City, California, USA), and analyzed on a 3130 Genetic Analyser sequencing machine (Applied Biosystems). Sequences were analyzed in silico for mutations using Blast (https://blast.ncbi.nlm.nih.gov/Blast.cgi).

### Mutation burden analysis

Genes biologically related to cilia were obtained from SYScilia database [67] (https://.Syscilia database.com). These genes were separated into two subsets of genes regarding their involvement in either primary or in motile cilia (Supplementary Table 5). The coverage of the selected genes in the exome was above 70% (exon_coverage_20x) except for *SHH* with a respective coverage of 59%. As control genes for permutations tests, 1,926 housekeeping genes identified in at least seven different studies (detective breadth ≥ 7; [107]) were used.

WES data from 25 unrelated hydrocephalus patients and 166 in-house controls, composed of patients presenting pathologies other than cerebral and their relatives, were used for the genetic mutation burden test analysis.

Population stratification of unrelated hydrocephalus patients and control patients was determined by principal component analysis (PCA) using PLINK software [108]. Mahalanobis distance (MD) with a significance level of 5% was used to identify potential outliers.

A genetic mutation burden test was used to assess if there was a significant excess of variants in ciliary genes in our patients compared to controls. WES data of controls and cases were analyzed to search for variants in genes related to ciliary structure. Variants were filtered for quality criteria (pass GATK [102] standard filter, read depth ≥ 10), AF (< 30%, 10%, 5%, 3%, 1%, 0.5% based on the maximum minor allele frequency found in ExAC [36], 1000G [37], ESP650 [39], GoNL [38], ARIC5606 [104], and our in‐house database) and mutation impact using snpeff_effect [106].

The genetic burden was analyzed using an in-house developed program in Python (https://www.python.org/). Statistical significance was measured by comparing the genetic burden of our patients to controls using a nonparametric Wilcoxon test. Precisely, for each patient or control, the number of allelic variants in ciliary genes was counted, with homozygous variants counting as two allelic variants. A permutation test with 10,000 random selections of 304 or 253 housekeeping genes was performed to exclude the effect of chance in all/primary cilia gene selection, respectively. A mutation burden was measured and the Wilcoxon statistic for independent samples was calculated for each of the 10,000 selections. The number of subsets of housekeeping genes, yielding a Wilcoxon statistic with a smaller p value than for the ciliary genes, was counted and divided by 10,000. This value was set as the p value for the permutation tests. An explanatory scheme is available in Additional file 1: Fig. S1.

### Predicting disease-causing variant combinations using ORVAL platform

Prediction of potentially disease-causing combinations was performed using VarCoPP [70, 109] on an in-house cluster. VarCoPP is designed to process alleles in pairs to prioritize disease-causing combinations. This classifier, trained on digenic cases contained in the digenic disease database (DIDA) [110], uses 11 features at the variant (e.g., CADD raw scores), gene (e.g., haploinsufficiency) and gene-pair level (e.g., biological distance). Specifically, 500 random forest predictors constitute VarCoPP, where each individual predictor classifies a given variant combination. Two scores are assigned to each combination, the classification score CS (i.e., median probability calculated over all the pathogenic probabilities provided by the ensemble of predictors) and the support score SS (i.e., percentage of the 500 predictors that deem the combination pathogenic). Thresholds are defined with regard to these two scores to create confidence zones. We considered bi-locus variant combinations that fells in the 99% confidence zone (CS ≥ 0.74; SS = 100%). These combinations were further inspected using the ORVAL plateform (https://orval.isquare.be) [70], which incorporates VarCoPP [109].

## Supplementary Information

**Additional file 1**. Patient data and ciliary methodology.

## Data Availability

The datasets used and/or analyzed during this study are available from the corresponding author on reasonable request.
