# Supplementary material for: Congenital hydrocephalus: new Mendelian mutations and evidence for oligogenic inheritance
Source: Hum Genomics. 2023 Mar 2;17:16. doi: 10.1186/s40246-023-00464-w (PMC9979489; doi:10.1186/s40246-023-00464-w)
Supplement: Supplementary file 1 — Additional file 1. Patient data and ciliary methodology. [file 40246_2023_464_MOESM1_ESM.docx]

## Supplementary Data

| **Family** | **Patient** | **Sex** | **Ethnicity** | **Consanguinity** | **Dysmorphism** | **Neurological anomalies** | **Laterality Defect (Primary Cilia)** | **Other congenital anomalies** | **MRI protocol** |
| --- | --- | --- | --- | --- | --- | --- | --- | --- | --- |
| 1 | 1.1 | F | Moroccan | n/a |  | severe encephalopathy |  | Severe intellectual deficiency | SA stenosis |
|  | 1.2 | F |  |  |  |  |  |  |  |
| 2 | 2 | M | Somalian | n/a | Macrocephaly (HC at 8months 55.5cm (>P97)), divergent strabism | severe axial and peripheral hypotonia, Parinaud phenomena | + |  | THC, DPS, temporal cyst |
| 3 | 3 | M | Moroccan | yes |  |  |  |  | THC |
| 4 | 4 | M | French | no | thin, pointed nose, low, poorly hemmed ears |  |  |  |  |
| 5 | 5 | M | Moroccan | yes | HC at birth 44.5cm, macrocephaly, microretrognathia | Optic nerve hypoplasia | + |  | SA stenosis, cerebral parenchyma atrophy, CC is thin and stretched, absence of SP, malformation of vertebrae |
| 6 | 6.1 | n/a | French | no |  |  | + |  | THC, SA stenosis, CC agenesis |
|  | 6.2 | n/a |  |  |  |  |  |  | THC |
| 7 | 7 | F | Belgian | no |  |  |  |  |  |
| 8 | 8 | M | Moroccan | no |  | mild axial hypotonia, epilepsy in spectrum of Lennox-Gastaut syndrome | + | Amniotic band amputation | SA stenosis, CC agenesis, Polymicrogyria, |
| 9 | 9 | F | Australian | no |  |  |  |  |  |
| 10 | 10 | n/a | Lebanese | yes |  |  |  |  |  |
| 11 | 11 | F | Chinese | yes |  |  | + | hypospade, intestinal malrotation, renal cysts | Quadriventricular HC, vermis hypoplasia, CC agenesis |
| 12 | 12 | F | Belgian | no |  |  |  |  |  |
| 13 | 13 | n/a | French | n/a |  |  |  |  |  |
| 14 | 14 | n/a | French | no |  |  |  |  |  |
| 15 | 15.1 | M | Finnish | no | OFC 37cm+1SD |  |  |  | pachygyria |
|  | 15.2 | M |  |  | Macrocephaly OFC 40.5cm+3SD |  |  |  |  |
| 16 | 16.1 | M | Pakistani | yes |  |  |  |  |  |
|  | 16.2 | F |  |  |  |  |  |  |  |
|  | 16.3 | M |  |  |  |  |  |  |  |
| 17 | 17 | M | Lebanese | yes |  |  |  |  |  |
| 18 | 18 | M | Portuguese | no |  |  | + |  | SA stenosis, CC agenesis |
| 19 | 19 | n/a | Italian | no |  |  |  |  |  |
| 20 | 20 | F | English | no |  |  |  |  | CC hypotrophy |
| 21 | 21 | M | French | no |  |  | + | anal imperforation, absence of pulmonary segmentation, cardiopathy, bilobate thymus, unique ombilical artery, enlarged renal bassinet | THC, SA stenosis, CC agenesis hypoplasia of bulbaire pyramids |
| 22 | 22 | F | Belgian | no | Pear-shaped head |  | + |  | Enlarged lateral ventricles, hemicerebellum hyploplasia, small cerebellum, cortical atrophy, CC thin and stretched, globally smaller brain, cyst on midline (supra and infra tentoriel), encephalocele |
| 23 | 23.1 | M | French | no |  |  | + |  | THC, CC agenesis |
|  | 23.2 | M |  |  |  |  |  |  |  |
| 24 | 24 | n/a | Armenian Turkish | yes |  |  |  | Unique umbilical artery, intraventricular communication, malrotation, accessory spleen, interarticular communication | Bilateral HC, small cerebellum |
| 25 | 25 | M | Belgian | yes |  |  |  |  |  |
| 26 | 26 | F | Belgian | no |  |  |  |  | H severe at 22weeks, small cerebellum, SA agenesis, mild hypoplasia vermis and cerebellum |
| 27 | 27 | M | Belgian | no |  |  |  |  |  |
| 28 | 28.1 | M | Turkish | yes |  |  |  |  | SA dysplasia, absence of bulbaire pyramids and CC |
|  | 28.2 | M |  |  |  |  |  | intrautrerine growth restriction (femur < 3er percentile) | Cerebellum hypoplasia, enlarged lateral ventricles, atrophy of cerebral parenchyma |

**Supplementary table 1 Clinical and radiological features in CH cohort (35 patients, 28 families)**

THC, triventricular hydrocephalus ; H, hydrocephalus; DPS, dilation of pericerebellar spaces; SA, Sylvius aqueduct; SP, septum pellucidum; CC, corpus callosum; HC, head circumference.


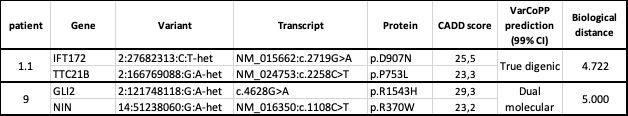


**Supplementary table 2 Candidate digenic pair in primary cilia genes**

Analysis of individual data in primary cilia genes revealed a true digenic pair as candidate digenic pairs for one patient. Combined Annotation Dependent Depletion (CADD) scores, ORVAL prediction in the 99% confidence interval, and Biological distance. A CADD score between 0-10 is associated with non-deleterious variants, and scores greater or equal to 20 are associated with the 1% most deleterious substitutions possible. VarCoPP predicts the pathogenicity of any bi-locus variant combination using a variant list from a single individual. The Biological distance is one of the 11 parameters taken into account by VarCoPP in pathogenicity predictions.

| **Inclusion criteria** |
| --- |
| Congenital hydrocephalus (prenatal or neonatal diagnosis) of unknown origin either isolated or associated with brain malformations including encephalocele, lissencephaly, and corpus callosum agenesis/hypoplasia. |
| **Exclusion criteria** |
| Demonstrated or highly suspected intrauterine infection during pregnancy  (including toxoplasmosis, rubella, cytomegalovirus, herpes virus, syphilis …)  Cocaine or other recreation drug use during pregnancy  Hydrocephalus due to intracerebral hemorrhage  Intracerebral tumor  Neural tube defect: Spina bifida/myelomeningocele  Craniosynostosis  Abnormal karyotype – pathological CNV in aCGH  Known mutation in L1CAM gene |

**Supplementary table 3 Inclusion and exclusion criteria for cohort selection.**

Cohort patient selection was based on inclusion and exclusion criteria as listed. CNV : copy number variant ; aCGH : comparative genomic hybridization array.

| **Patient ID** | **Genes** | **Primers (5’-3’)** | |
| --- | --- | --- | --- |
| 10 | TIE1 | For | ACTGAAACCTCCTCGTGTGC |
|  |  | Rev | CTGGCAAGCTACTCATGTGG |
| 21 | ARID1A | For | GCATCCAGGACAACAATGTG |
|  |  | Rev | TTTCTAAGTTCTCCACACACGC |
| 27.1 and 27.2 | RNPC3 | For | tgacctcttctactgatGCATTG |
|  |  | Rev | TCTCTAAAAGGAAAATGATGGG |

**Supplementary table 4 Primers used for Sanger Sequencing.**

**Supplementary table 5 Ciliary gene list.**

Ciliary gene list adapted from SYScilia database v1 and further curated in primary and motile cilia genes


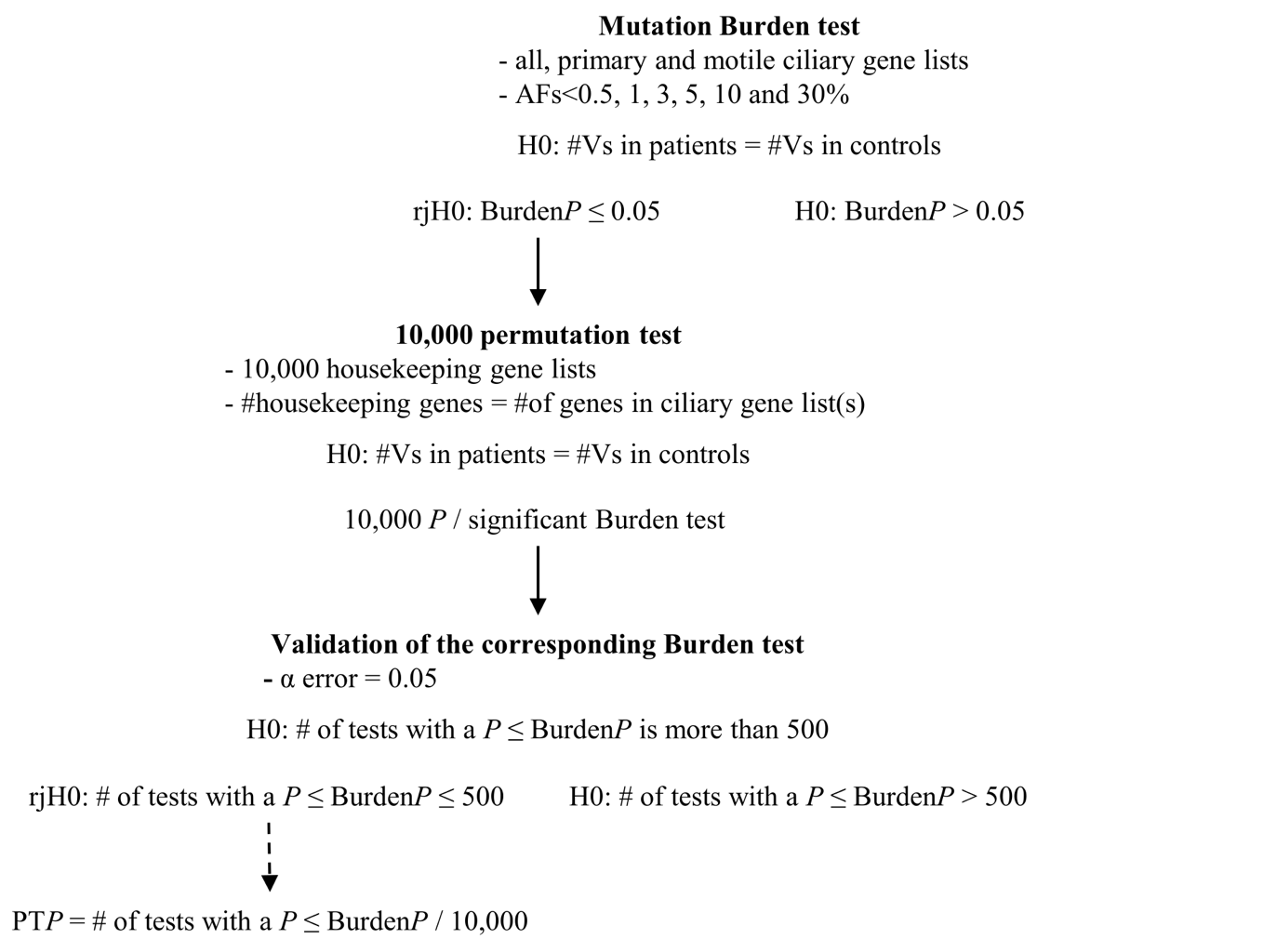


**Supplementary figure 1 Statistical analysis of burden test and their validation**

Burden tests were performed for each ciliary gene list at different allelic frequencies (AFs) using a Wilcoxon test to compare the number of variants (#Vs) in patients and controls. For tests with a significant p-value, a permutation test (PT) was performed in the same conditions as the considered test (i.e. number of genes and AF). The PT  is composed of 10,000 burden tests run on randomly chosen housekeeping genes. Specifically, 10,000 Wilcoxon tests compare #Vs in patients and controls for a number of housekeeping genes equal to the number of genes included in the considered ciliary gene list at a particular AF. To validate the considered Burden test, the number of tests harboring a p-value smaller than the p-value obtained in the significant Burden test are counted. As an α error = 0.05 is considered, the number of tests with a smaller p-value than the one considered must be less than 500 (10,000 * 0.05) to validate the Mutation Burden test. Finally, the number of tests was divided by 10,000 and set as the permutation test p-value (PT*P*). AFs, allelic frequencies; #, number; #Vs, number of variants; H0, null hypothesis; rjH0, reject of H0; *P*, p-value;  PT*P*, permutation test p-value


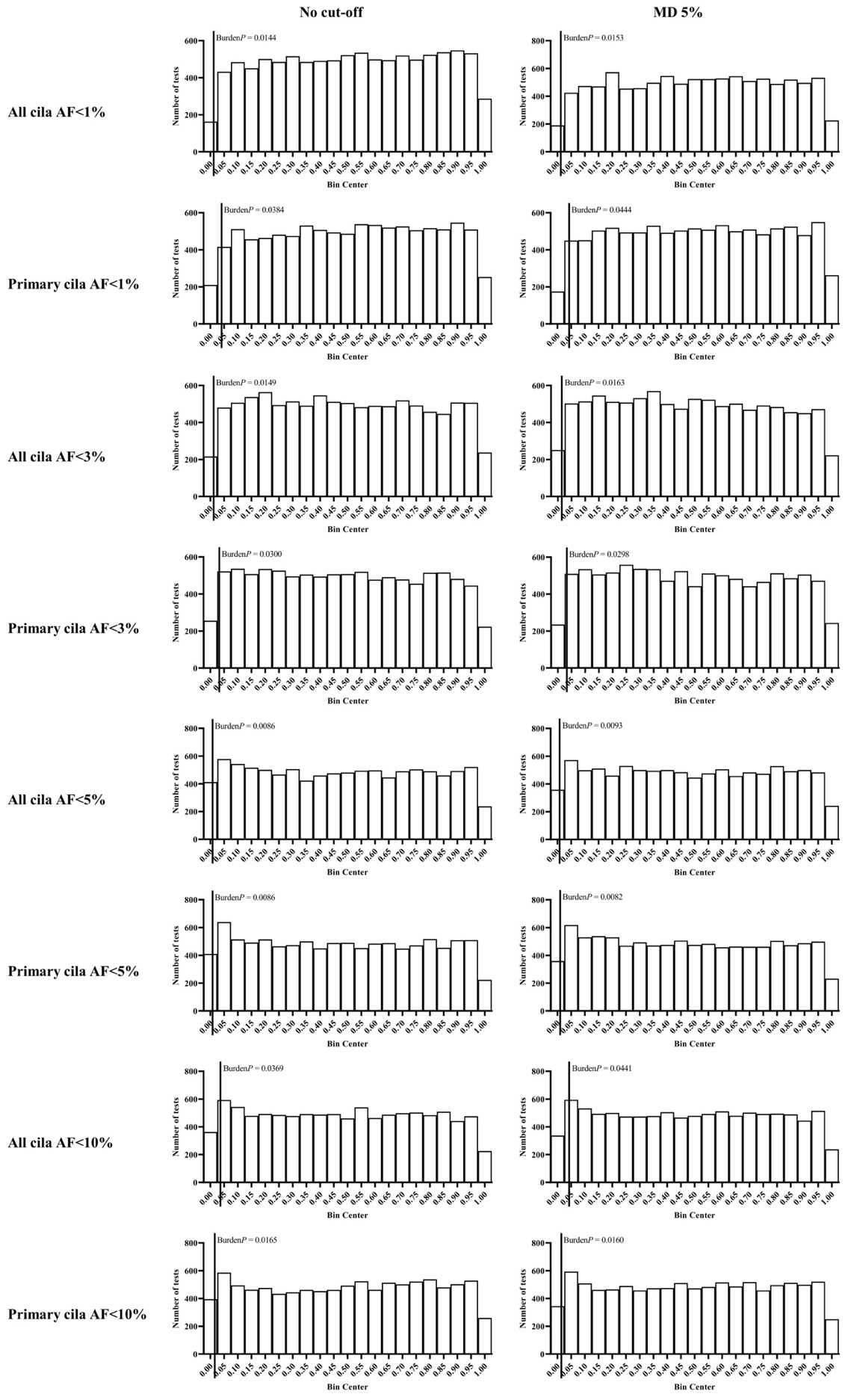


**Supplementary figure 2 Frequency distribution of the 10,000 permutation tests.**

Bar graph which represents all the p-values obtained in the 10,000 permutation tests (PT) for the ciliary subsets, allelic frequencies (AF) and cut-offs, which gave a significant Burden test (Burden*P*). Each bin contains a number of values (p-values of the PT) that lie within the range of values that is defined by the bin width (0.05). The Burden*P* (vertical bar) defines the threshold considered to count the number of PT p-values which are smaller or equal to (left) or greater than (right) the threshold. The burden test is considered accepted if the number of p-value.
